# Satiety: a gut–brain–relationship

**DOI:** 10.1186/s12576-024-00904-9

**Published:** 2024-02-17

**Authors:** Ghinwa M. Barakat, Wiam Ramadan, Ghaith Assi, Noura B. El Khoury

**Affiliations:** 1 Biological and Chemical Sciences Department, School of Arts and Sciences, Lebanese International University, Beirut, Lebanon; 2 Nutrition and Food Sciences Department, School of Arts and Sciences, Lebanese International University, Beirut, Lebanon; 3 Gilbert and Rose-Marie Chagoury School of Medicine, Lebanese American University, Beirut, Lebanon; 4 Psychology department, Faculty of Arts and Sciences, University of Balamand, Balamand, Lebanon

**Keywords:** Satiety, Microbiota, GLP-1, Neurotransmitters, Neuroscience

## Abstract

Many hormones act on the hypothalamus to control hunger and satiety through various pathways closely associated with several factors. When food is present in the gastro intestinal (GI) tract, enteroendocrine cells (EECs) emit satiety signals such as cholecystokinin (CCK), glucagon like peptide-1 (GLP-1) and peptide YY (PYY), which can then communicate with the vagus nerve to control food intake. More specifically, satiety has been shown to be particularly affected by the GLP-1 hormone and its receptor agonists that have lately been acknowledged as a promising way to reduce weight. In addition, there is increasing evidence that normal flora is also involved in the peripheral, central, and reward system that impact satiety. Moreover, neurologic pathways control satiety through neurotransmitters. In this review, we discuss the different roles of each of the GLP-1 hormone and its agonist, gut microbiomes, as well as neurotransmitters and their interconnected relation in the regulation of body’s satiety homeostasis.

## Introduction

### Hormones involved in satiety

The hypothalamus is a brain structure that plays a major part in the complex neural network that controls the homeostatic regulation of energy balance [1]. It is the center for all hormones involved in the satiety mechanism regulation. Of these hormones are peripheral anorexigenic hormones that improve satiety such as glucagon like peptide -1 (GLP-1), peptide YY (PYY), insulin, cholecystokinin (CCK) and leptin. Even ghrelin, which is a hormone that stimulates hunger, acts on hypothalamus [2]. It is known that central Pro-opiomelanocortin (POMC) and cocaine–amphetamine-regulated transcript-containing (CART) neurons in the hypothalamus increase satiety, while neurons carrying neuropeptide Y (NPY) and agouti-related peptide (AgRP) trigger the desire to eat [3]. The hypothalamus also transmits signals to the mesolimbic rewards circuit, which is crucial for addiction, impulsive behavior, and food reward [4]. Signaling of the peripheral hormones mentioned earlier have been even connected to impulsivity [5] and addiction [6]. This emphasizes further the role of physiological and/or psychological factors in eating disorders [7].

### Microbiota role in metabolism

Over the last decade, there has been an increasing interest in the role of the gut microbiota in the physiology of both health and disease [8]. These multitudes of intestinal inhabitants collaborate with the host in a crucial evolutionary relationship to preserve homeostasis [9]. A growing body of evidence demonstrates that the gut microbiota has a substantial impact on the bidirectional connection between the GI tract and the brain, known as the microbiota–gut–brain axis [10]. Particularly, it is becoming more recognized that the host's microbiome affects how efficiently it uses its own energy, which can lead to metabolic and eating disorders [11]. For instance, changing gut microbiota composition has been linked to anorexia nervosa [12] and obesity [13].

### Neuroscience in relation to satiety

The arcuate nucleus of the hypothalamus is the region that seems to be most crucial in the integration of signals about energy flux. It gets signals related to immediate satisfaction (satiety related to the early stages of digestion, particularly in the stomach and initial parts of the digestive system) that interact with signals derived from adiposity. POMC, a precursor for a number of peptides like α-melanocyte stimulating hormone (α-MSH), endorphins, and adrenocorticotropic hormone (ACTH), is expressed by melanocortin system neurons in the arcuate nucleus [14]. Melanocortin 4 receptor (MC4R), the main receptor for α -MSH, is found in the arcuate nucleus as well as various other parts of the brain [15]. When α-MSH or an agonist binds to the MC4R, catabolic pathways are triggered, resulting in hypophagia, thermogenesis, and weight loss [16], whereas MC4R antagonists cause weight gain and hyperphagia [17].

## Body

### Hormones and satiety-GLP-1

#### GLP-1 and satiety

GLP-1 is an incretin hormone made by the L cells of the intestine. GLP-1 receptors are abundant in the arcuate nucleus as well as the hypothalamus which contains projections to the hunger centers [18, 19]. By its peripheral and central activities, GLP-1 decreases calorie intake, boosts feelings of satiety, and encourages weight loss [20]. It was shown that food intake is inhibited by acute intracerebroventricular GLP-1 injection, and food intake is increased—even in satiated rats—by antagonists to the GLP-1 receptor [21]. The strong evidence that the paraventricular nucleus is the principal site for brain-derived GLP-1 satiety comes from the direct delivery of GLP-1 into this region of the brain. GLP-1 exerts its effects by acting directly on the paraventricular nucleus. Yet, because POMC neurons express GLP-1 receptors, GLP-1 also has anorexigenic actions in the arcuate nucleus [22]. Neuronal circuits are activated, and food intake declines as satiety signals like CCK and GLP-1 are created during food ingestion, signaling the conclusion of the meal. Finally, GLP-1 injections into the body over an extended period of time decrease weight gain and promote weight loss [23]. This is also supported by the observation that obese people have lower GLP-1 levels than lean people [24].

#### GLP-1 agonist effect on microbiota

The GLP-1 receptor agonist liraglutide has lately been acknowledged as a promising anti-obesity medication in obese and/or diabetic people [25]. The literature demonstrated that alterations in gut microbiota also significantly impacted satiety, lipid metabolism, and ectopic fat deposition, through the effect of GLP-1or its agonists. For instance, liraglutide, may thereby prevent weight gain via modifying the composition of the gut's microbial population [26]. More specifically, a previous study showed that liraglutide can, in fact, alter the makeup of the gut microbiota by boosting the lean-related profile, which is consistent with its ability to reduce body weight in mice with streptozotocin-induced transient hyperglycemia [27]. Similarly, liraglutide was found to reduce weight gain in both diabetic and nondiabetic obese patients by altering the composition of the gut flora [28]. According to another study, liraglutide causes gut microbial structural alterations in diet-induced obese (DIO) mice, with the distribution of *Proteobacteria* and *Verrucomicrobia* phylotypes changing the most, while *Firmicutes* remain relatively unaffected [29]. The reduction in *Proteobacteria* lead to a drop in total body mass and the adiposity index, which were indicators of decreased food intake and feeding effectiveness [29]. Since *Verrucomicrobia* support the human gut’s glucose balance, its reduction will disrupt glucose homeostasis and therefore satiety [30]. Interestingly, it has been suggested that *Firmicutes*, a phylum that produces a significant amount of short chain fatty acids (SCFAs), particularly butyrate, may contribute to host obesity by enabling weight gain mechanism such as increase nutrition processing and energy extraction [31]. This can explain why GLP-1 does not stimulate *Firmicutes* as they both have opposite outcomes. Moreover, the abundance of *Akkermansia muciniphila*, a species known to degrade mucin and produce SCFAs, was found to be positively correlated with indicators of gut inflammation and significantly associated with body weight loss when its proportion increase due to liraglutide administration [32]. Another study suggested that the changes in the microbiome may be related to the GLP-1 and receptor signaling's convergent physiologic effects on calorie intake, glucose metabolism, and lipid management [33].

### Microbiota in relationship to satiety

#### Microbiome and peripheral satiety mechanism

The story of the interaction between the gut microbiome and the neural circuits to the brain starts when food is ingested where CCK, GLP-1 and PYY are secreted by enteroendocrine cells (EECs) in order to send satiety signals via the vagus nerve in the purpose to control food intake [34]. There are many studies that reveal a “gut–brain” communication which is modulated by the gastrointestinal (GI) bacterial composition as we will mention in the following:

#### Microbiota and CCK

A person's gut microbiota may modify the expression and release of GI satiety peptides, which in turn may impact how much they eat. This idea was demonstrated in rodents. For instance, studies show that when compared to typical mice of the same weight, germ-free (GF) mice (that lack microbiota) exhibit reduced intestine expression of the CCK peptide [35]. Although the role of food receptors in the increased caloric intake seen in GF mice is unknown, it is suggested that the activation of nutrient responsive receptors triggers the release of satiety peptides from the intestinal tract, including CCK [36]. This indicates that these receptors play a role through the regulation of satiety peptides availability. For example, in cases of fructose malabsorption, there are higher relative abundances of *Actinobacteria*, *Bacteroidetes*, and *Lactobacillaceae* (especially *Lactobacillus johnsonii*). This indicates that fructose malabsorption induces CCK expression into the intestine by changing microbiota composition and metabolism. These adjustments are followed by a large rise in the number of CCK-positive enteroendocrine cells (EECs), proving that fructose malabsorption-induced changes in CCK release require the microbiota [37]. Moreover, increased CCK release is seen in the murine EEC line STC-1 when specific fatty acid metabolites generated by colonized lactic acid bacteria are applied [38]. All these data suggest that the relation between CCK and microbiota is thought to be due to microbial-derived products like lipopolysaccharide (LPS) and metabolites such SCFA [39] which act on enteroendocrine cells to release CCK.

#### Microbiota and GLP-1

The impact of the microbiome on gut satiety peptides extends beyond CCK. GLP-1 is secreted from intestinal L-cells and reduces appetite through a vagal-mediated mechanism [40]. It was found that intestinal bacteria ferment the prebiotic fiber beta-glucan to generate propionate, one of the important SCFAs [41]. Evidence suggests that a healthy microbiome's production of SCFAs affects the release of GLP-1. In a study conducted by Tolhurst and his colleagues, the free fatty acid receptor (FFAR 2), a nutrient-sensing G-protein coupled receptor, is activated when SCFAs—acetate, propionate, and butyrate—are applied to mouse colonic cell cultures. The activation of (FFAR 2) receptor resulted in an increase in GLP-1 production. Hence, the interaction of bacterial metabolites like propionate with intestinal L-cells can directly control GLP-1 production [42]. Moreover, according to recent research, acute inulin-propionate ester supplementation elevated plasma GLP-1 and PYY levels. It was linked to lower food consumption at meals after supplementation in humans. This demonstrates the immediate impact of propionate on meal consumption [43], and shows how microbial product help in physiologic control of satiety hormonal release. Also, it is interesting to note that prebiotic supplementation increases colon mass in mice when compared to non-supplemented controls [44], which can be partially attributed to an increase in the number of secretory cells [45]. Another way that the microbiota may affect GLP-1 release is through metabolites. For example, levels of acyl-glycerols in the gut are restored in obese mice when *Akkermansia muciniphila* is administered as a probiotic [46] as mentioned in Table 1. Acylglycerols, which are byproducts of fat digestion, activate a G-protein-coupled receptor, which in turn prompts L cells to release gut peptides including GLP-1 [47].

**Table 1:** Main mechanisms by which microbiota affect satiety control

Lactobacillus rhamnosus, Lactobacillus acidophilus, Bifidobacterium bifidum	Decrease food intake and hypothalamic inflammation [50]
Akkermansia muciniphila	Enhance gut peptide release though increasing acyl-glycerols in the gut [46]
Gram-negative bacteria	Produce LPS which can reduce hypothalamic inflammation and body weight [52]
Proteus mirabilis	Decrease vagal afferent neuron (VAN) survival [53]

#### Microbiota and central satiety mechanism: satiety and neurological inflammation

As mentioned above, the hypothalamus is home to important anorexigenic and orexigenic neuronal populations that control hunger and energy expenditure. Leptin in particular can alter the expression and release of neuropeptides in the hypothalamus to control energy homeostasis.

The hypothalamus and the nucleus of tractus solitarius (NTS) have been related to inflammation which lead to loss of function as a result of bacterial inflammatory agents generated by the obese-type microbiome [48]. Leptin sensitivity in neurons is compromised by inflammation and cytokine signaling [49]. Particularly, leptin can alter the expression and release of neuropeptides in the hypothalamus to control energy homeostasis. Thus, affecting leptin sensitivity can disturb energy homeostasis in the hypothalamus. In diet-induced obesity (DIO) mice, taking a probiotic supplement containing Lactobacillus rhamnosus, *Lactobacillus* acidophilus, and Bifidobacterium bifidum reduces body weight and food intake. It also normalizes and restores leptin-induced phosphorylated signal transducer and activator of transcription 3 (pSTAT3) expression (Table 1**)** [50]. *Lactobacillus rhamnosus* supplementation alone has resulted in identical preservation of leptin signaling, proving that hypothalamic leptin signaling is affected by the presence of specific bacteria [51].

On the other hand, a study In diet-induced obesity (DIO) mice, taking a probiotic supplement containing Lactobacillus rhamnosus, Lactobacillus acidophilus, and Bifidobacterium bifidum reduces body weight and food intake. It also normalizes and restores leptin-induced pSTAT3 expression [50]. *Lactobacillus rhamnosus* supplementation alone has resulted in identical preservation of leptin signaling, proving that hypothalamic leptin signaling is affected by the presence of specific bacteria [51].

In brief, the microbiome plays an important role in controlling central satiety metabolism and this role is mediated by the release of different cytokines.

#### Microbiota and reward system

In addition to the role of neurologic inflammation as central mechanism, brain reward system can also alter satiety.

Food accessibility, social and environmental cues, and flavor are a few examples of external influences that might interfere with the body’s natural intake control [40]. The dorsal striatum's dopamine (DA) levels are sufficiently raised by optogenetic activation of vagal afferent neurons (VANs) that innervate the upper GI tract to promote reward-related behaviors such self-stimulation, location preference, and flavor conditioning [34].

Antibiotic-free and germ-free mice show changes in dopaminergic reward pathways [54]. Contrary to conventional mice, GF mice had greater desire for even low concentrations of intralipid [35]. GF mice showed enhanced DA turnover in the striatum and reduced expression of D1 receptor mRNA in the striatum and nucleus accumbens (NAc) [55], two areas implicated in food-seeking behavior [56]. This shows that in situations where dopamine is high, people tend to have higher desire to eat. Antimicrobial therapy elevated L-3,4-dihydroxyphenylalanine (L-DOPA) in young mice’s amygdala and lowered DA turnover in rats' amygdala and striatum, indicating that the microbiome regulates DA neurochemistry [57]. Adolescent rats with periodic daily access to the high fat/high sucrose (HFHS) diet have higher total energy expenditure and changed monoamine gene expression in the hippocampus and prefrontal cortex. One of the changes observed is related to the monoamine oxidase A (MAO-A) that is an enzyme involved in removing the neurotransmitters norepinephrine, serotonin and dopamine from the brain. These changes are correlated with bacterial distribution and abundance. In particular, MAO-A expression in the hippocampus is linked to several other bacterial genus including unspecified Bifidobacteriales, Bifidobacteriaceae, and an unspecified genus of the *Lachnospiraceae* family. In contrast, MAO-A expression in the prefrontal cortex is positively linked to an unspecified genus of the *Lachnospiraceae* family [58].

Food preferences could be influenced by microbiota exposed to particular conditions. For instance, mice under social stress show increased preference for sucrose, and this preference is eliminated by SCFA supplementation, suggesting that the microbiota controls stress-induced sucrose preference through the synthesis of SCFA [59].

Also, artificial sweeteners with low or no calories are another topic of concern in terms of how they affect intake and satisfaction. This is because it has been shown that some sweeteners, like stevia, are digested by gut flora [60]. Although the consumption of artificial sweeteners does not appear to trigger compensatory overeating in humans in short-term or long-term trials, it has been shown to modify reward circuits in both rodents and humans [61]. For instance, tyrosine hydroxylase and dopamine transporter (DAT) mRNA expression in the NAc is reduced in rats exposed to a chronic low dosage of the stevia glycoside rebaudioside A (RebA), which can be reversed by supplementing with the prebiotic oligo-fructose [62]. These findings imply that the metabolism of artificial sweeteners by bacteria may change reward signaling and that the rewarding qualities of food might override the basic satiety signals produced by homeostatic regions [63].

In conclusion, the presence of microbiota leads to the activation of the reward system which thus increases food desire, whereas their absence can depress reward system and reduce food desire.

### Neuroscience in relation to satiety

Many studies have shown that the homeostatic regulator of food intake interacts with the dopamine reward system leading to a boosting effect on food intake. This interaction is based on the involvement of the dopamine reward system in the behavior of food seeking [64]. For instance, it has been shown that ghrelin stimulates ventral tegmental area (VTA) dopamine neurons whereas leptin and insulin inhibit them [65]. According to research by Hommel et al., leptin receptors are expressed on VTA dopamine neurons and inhibit their activity. Food intake was observed to decrease when leptin was administered to the VTA, but it increased when leptin receptors were knocked down in the VTA, along with activity levels and hedonic feeding [66].

#### Neurotransmitter effect on satiety

In the parabrachial nucleus, a region located in the pons, the neurotransmitter gamma amino benzoic acid (GABA) produced by NPY and AgRP neurons, maintains energy balance [67]. The dorsal raphe nucleus (DRN) contains a population of heat-activated GABAergic neurons that control energy expenditure via altering motility and thermogenesis [68]. The increase in motility and thermogenesis lead to increased desire to eat and replenish energy sources. Its significance in obesity is clear from the fact that eliminating the vesicular transporter for GABA in AgRP neurons causes resistance to obesity brought on by a high-fat diet, regardless of changes in food intake [69]. Serotonin receptor, which is found in certain arcuate POMC neurons, is another neurotransmitter that controls how much food is consumed and how much energy is expended [70]. Independently of changes in energy expenditure, these POMC serotonin receptors are engaged in controlling energy homeostasis through changes in eating behavior [71] as shown in Table 2. Through the MC4R sympathetic preganglionic neurons, POMC neurons that project to the spinal cord are also engaged in maintaining homeostasis of energy by promoting adaptive thermogenesis in brown adipose tissue [72]. As a result of energy expenditure, the desire to eat will also expand. Moreover, due to its role in maintaining energy homeostasis, oxytocin, a centrally acting neurotransmitter and hormone, is receiving more attention as a potential anti-obesity target [73]. Obesity was shown to be a characteristic of mice lacking either oxytocin or oxytocin receptors [74]. Also, in diet-induced obesity and genetically obese mouse models, long-term peripheral or central administration of oxytocin causes an inhibition of food intake, an increase in energy expenditure, and weight loss as seen in Table 2 [75].

**Table 2:** Different effects of neurotransmitters on satiety mechanisms and outcome

α-MSH	Catabolic pathways are triggered, resulting in hypophagia, thermogenesis, and weight loss [16]
Dopamine	Ghrelin stimulates ventral tegmental area (VTA) dopamine neurons and food intake whereas leptin and insulin inhibit them (low food intake) [65]
GABA	Low GABA resistance to obesity brought on by a high-fat diet [69]
Serotonin	Engaged in controlling energy homeostasis through changes in eating behavior [71]
Oxytocin	An inhibition of food intake, an increase in energy expenditure, and weight loss [75]

## Conclusion

The gut microbiota, which is the term for the whole microbial community inhabiting the digestive system, has been shown in several studies to be influenced by GLP-1. By encouraging the development of specific advantageous bacteria in the gut, GLP-1 may make it easier to produce satiety related microbial products. On the other hand, GLP-1 shortage or resistance may cause dysbiosis, or an imbalance of harmful and helpful microbes, which can exacerbate metabolic diseases like obesity and insulin resistance.

In conclusion, each of the hormonal, microbial and neurotransmitters are important for controlling satiety and glucose metabolism. Moreover, ongoing research on the connection between GLP-1 and microbiota could yield new insights and treatments for metabolic disorders.

## Data Availability

Lebanese Internation University (our instituation) funds publishing this article.
